# Supplementary material for: Proteomic Profile of M. longissimus thoracis from Commercial Lambs Reared in Different Forage Systems
Source: Foods. 2022 May 13;11(10):1419. doi: 10.3390/foods11101419 (PMC9141604; doi:10.3390/foods11101419)
Supplement: Supplementary file 1 [file foods-11-01419-s001.zip › foods-1687966-supplementary-done.pdf]

foods

MDPI

Table S1. List of total identified proteins in *longissimus lumborum* muscle from six types of typical commercial New Zealand forage lamb production systems.

Accession Number	Significance	Coverage (%)	Total Peptides	Unique Peptides	Post-translational modification	Description
XP_011992886.2	1.63	12	78	77	Carbamidomethylation; Deamidation (NQ); Pyro-glu from Q	LOW QUALITY PROTEIN: nebulin [Ovis aries]
XP_027824589.1	6.88	41	76	69	Carbamidomethylation; Deamidation (NQ); Phosphorylation (STY); Dehydration; Dethiomethyl	LOW QUALITY PROTEIN: filamin-C [Ovis aries]
W5PX04|W5PX04_SHEEP	2.24	49	50	49	Carbamidomethylation; Deamidation (NQ); Oxidation (M); Phosphorylation (STY); Dehydration; Pyro-glu from Q	Myosin binding protein C fast type OS=Ovis aries OX=9940 GN=MYBPC2 PE=4 SV=1
XP_027815274.1	1.36	72	63	47	Carbamidomethylation; Deamidation (NQ); Oxidation (M); Dehydration; Pyro-glu from Q	alpha-actinin-3 isoform X2 [Ovis aries]
XP_011957551.3	1.36	69	63	47	Carbamidomethylation; Deamidation (NQ); Oxidation (M); Dehydration; Pyro-glu from Q	alpha-actinin-3 isoform X1 [Ovis aries]
sp|O18751|PYGM_SHEEP	1.4	48	45	36	Carbamidomethylation; Deamidation (NQ); Oxidation (M); Pyro-glu from Q; Formylation	Glycogen phosphorylase muscle form OS=Ovis aries OX=9940 GN=PYGM PE=2 SV=3
XP_012036281.1	4.43	60	34	33	Carbamidomethylation; Deamidation (NQ); Oxidation (M); Dehydration; Pyro-glu from Q; Formylation	pyruvate kinase PKM isoform X1 [Ovis aries]
XP_004021419.1	1.76	57	53	33	Carbamidomethylation; Deamidation (NQ); Oxidation (M); Pyro-glu from Q	alpha-actinin-2 [Ovis aries]
XP_012045938.1	4.26	80	59	31	Carbamidomethylation; Deamidation (NQ); Oxidation (M); Phosphorylation (STY); Dehydration; 2 more	LOW QUALITY PROTEIN: creatine kinase M-type [Ovis aries]
XP_014959134.2	1.82	22	28	28	Carbamidomethylation; Deamidation (NQ); Oxidation (M); Pyro-glu from Q	myomesin-1 isoform X1 [Ovis aries]
W5QDF3|W5QDF3_SHEEP	2.87	49	107	26	Carbamidomethylation; Deamidation (NQ); Oxidation (M); Phosphorylation (STY); Dehydration; 3 more	Myosin heavy chain 7 OS=Ovis aries OX=9940 GN=MYH7 PE=3 SV=1
XP_004010374.1	2.87	49	107	26	Carbamidomethylation; Deamidation (NQ); Oxidation (M); Phosphorylation (STY); Dehydration; 3 more	myosin-7 [Ovis aries]
XP_012040978.1	0.33	73	40	26	Carbamidomethylation; Deamidation (NQ); Oxidation (M); Phosphorylation (STY); Dehydration	beta-enolase isoform X1 [Ovis aries]

XP_012032382.1	4.02	26	25	25	Carbamidomethylation; Deamidation (NQ)	glycogen debranching enzyme [Ovis aries]
NP_001177319.1	3.31	74	30	24	Carbamidomethylation; Deamidation (NQ); Oxidation (M); Dehydration	glyceraldehyde-3-phosphate dehydrogenase [Ovis aries]
W5PDG3|W5PDG3_SHEEP	3.31	73	30	24	Carbamidomethylation; Deamidation (NQ); Oxidation (M); Dehydration	Glyceraldehyde-3-phosphate dehydrogenase OS=Ovis aries OX=9940 GN=GAPDH PE=3 SV=1
NP_001135988.1	1.05	60	23	23	Carbamidomethylation; Deamidation (NQ); Oxidation (M); Pyro-glu from Q	phosphoglycerate kinase 1 [Ovis aries]
XP_004006621.1	0.88	62	22	22	Deamidation (NQ); Oxidation (M)	ATP synthase subunit beta mitochondrial [Ovis aries]
XP_004005001.1	1.95	51	24	21	Carbamidomethylation; Deamidation (NQ); Pyro-glu from Q	desmin [Ovis aries]
XP_004006413.2	2.79	45	19	19	Carbamidomethylation; Deamidation (NQ); Oxidation (M)	glycerol-3-phosphate dehydrogenase [NAD(+)] cytoplasmic isoform X1 [Ovis aries]
W5PW05|W5PW05_SHEEP	2.36	45	19	19	Carbamidomethylation; Deamidation (NQ); Dehydration; Formylation	Malate dehydrogenase 2 OS=Ovis aries OX=9940 GN=MDH2 PE=4 SV=1
XP_004021309.2	2.36	54	19	19	Carbamidomethylation; Deamidation (NQ); Dehydration; Formylation	malate dehydrogenase mitochondrial [Ovis aries]
XP_004020913.1	1.01	31	28	19	Carbamidomethylation; Deamidation (NQ); Oxidation (M)	sarcoplasmic/endoplasmic reticulum calcium ATPase 1 isoform X1 [Ovis aries]
W5NVR1|W5NVR1_SHEEP	1.01	32	28	19	Carbamidomethylation; Deamidation (NQ); Oxidation (M)	Calcium-transporting ATPase OS=Ovis aries OX=9940 GN=ATP2A1 PE=3 SV=1
XP_004020912.1	1.01	31	28	19	Carbamidomethylation; Deamidation (NQ); Oxidation (M)	sarcoplasmic/endoplasmic reticulum calcium ATPase 1 isoform X2 [Ovis aries]
XP_027823736.1	0.89	76	19	19	Carbamidomethylation; Deamidation (NQ)	triosephosphate isomerase [Ovis aries]
XP_012030289.1	1.38	15	19	18	Carbamidomethylation; Deamidation (NQ); Oxidation (M); Dehydration; Formylation	myosin-binding protein C slow-type isoform X2 [Ovis aries]
XP_012030288.1	1.38	15	19	18	Carbamidomethylation; Deamidation (NQ); Oxidation (M); Dehydration; Formylation	myosin-binding protein C slow-type isoform X1 [Ovis aries]
XP_004009106.3	2.67	50	22	17	Carbamidomethylation; Deamidation (NQ); Oxidation (M); Phosphorylation (STY); Dehydration; Formylation	creatine kinase S-type mitochondrial [Ovis aries]
W5P323|W5P323_SHEEP	3.18	45	16	16	Deamidation (NQ); Oxidation (M)	Glucose-6-phosphate isomerase OS=Ovis aries OX=9940 GN=GPI PE=3 SV=1
XP_004015200.2	3.18	45	16	16	Deamidation (NQ); Oxidation (M)	glucose-6-phosphate isomerase [Ovis aries]
XP_004020569.1	0.59	37	16	16	Deamidation (NQ)	ATP synthase subunit alpha mitochondrial [Ovis aries]

A0A3Q9U3M0|A0A3Q9U3M0_SHEEP	4.03	25	16	14	Carbamidomethylation; Deamidation (NQ); Oxidation (M); Pyro-glu from Q	ATP-dependent 6-phosphofructokinase OS=Ovis aries OX=9940 GN=PFKM PE=2 SV=1
XP_004006455.2	4.03	25	16	14	Carbamidomethylation; Deamidation (NQ); Oxidation (M); Pyro-glu from Q	LOW QUALITY PROTEIN: ATP-dependent 6-phosphofructokinase muscle type [Ovis aries]
W5QDD4|W5QDD4_SHEEP	4.03	25	16	14	Carbamidomethylation; Deamidation (NQ); Oxidation (M); Pyro-glu from Q	ATP-dependent 6-phosphofructokinase OS=Ovis aries OX=9940 GN=PFKM PE=3 SV=1
XP_004002712.1	1.99	48	14	14	Deamidation (NQ); Dehydration	calsequestrin-1 [Ovis aries]
XP_014953428.1	1.04	66	14	14	Carbamidomethylation; Deamidation (NQ)	carbonic anhydrase 3 [Ovis aries]
XP_027823951.1	2.52	23	13	13	Deamidation (NQ); Pyro-glu from Q	aconitate hydratase mitochondrial [Ovis aries]
NP_001159670.1	1.53	46	13	13	Carbamidomethylation; Deamidation (NQ)	four and a half LIM domains protein 1 [Ovis aries]
XP_027818556.1	1.53	48	13	13	Carbamidomethylation; Deamidation (NQ)	four and a half LIM domains protein 1 isoform X3 [Ovis aries]
XP_027815619.1	0.57	61	17	13	Carbamidomethylation; Deamidation (NQ); Pyro-glu from Q	L-lactate dehydrogenase A chain isoform X1 [Ovis aries]
W5PDD8|W5PDD8_SHEEP	4.13	55	12	12	Deamidation (NQ)	Myozenin 1 OS=Ovis aries OX=9940 GN=MYOZ1 PE=4 SV=1
XP_027817273.1	3.8	83	12	12	Pyro-glu from Q	heat shock protein beta-1 [Ovis aries]
XP_004015091.1	3.94	29	10	10	Carbamidomethylation; Deamidation (NQ)	aspartate aminotransferase mitochondrial [Ovis aries]
XP_004020149.1	1.21	31	10	10	Carbamidomethylation; Deamidation (NQ)	aspartate aminotransferase cytoplasmic [Ovis aries]
XP_004004324.1	0.97	58	27	10	Carbamidomethylation; Dehydration; Pyro-glu from Q	tropomyosin beta chain isoform X1 [Ovis aries]
W5PVY5|W5PVY5_SHEEP	0.94	68	15	10	Carbamidomethylation; Deamidation (NQ); Oxidation (M)	Phosphoglycerate mutase OS=Ovis aries OX=9940 GN=PGAM2 PE=3 SV=1
NP_001155354.1	0.29	63	11	10	Carbamidomethylation; Deamidation (NQ); Oxidation (M)	adenylate kinase isoenzyme 1 [Ovis aries]
XP_011955483.2	2.01	27	9	9	Carbamidomethylation; Deamidation (NQ)	cytochrome b-c1 complex subunit 1 mitochondrial [Ovis aries]
XP_004004940.1	1.21	85	34	9	Carbamidomethylation; Deamidation (NQ); Oxidation (M); Phosphorylation (STY); Dehydration; 2 more	myosin light chain 1/3 skeletal muscle isoform isoform X1 [Ovis aries]
XP_004018103.4	0.86	25	9	9	Carbamidomethylation; Deamidation (NQ)	isocitrate dehydrogenase [NADP] mitochondrial [Ovis aries]

W5PR04|W5PR04_SHEEP	0.86	26	9	9	Carbamidomethylation; Deamidation (NQ)	Isocitrate dehydrogenase [NADP] OS=Ovis aries OX=9940 GN=IDH2 PE=3 SV=1
XP_027832152.1	0.73	80	10	9	Carbamidomethylation; Deamidation (NQ); Oxidation (M)	troponin C skeletal muscle isoform X1 [Ovis aries]
XP_004004668.2	0.6	12	9	9	Carbamidomethylation; Deamidation (NQ)	kelch-like protein 41 [Ovis aries]
W5NZL8|W5NZL8_SHEEP	0.6	12	9	9	Carbamidomethylation; Deamidation (NQ)	Kelch like family member 41 OS=Ovis aries OX=9940 GN=KLHL41 PE=4 SV=1
W5PI38|W5PI38_SHEEP	0.11	20	9	9	Carbamidomethylation	Citrate synthase OS=Ovis aries OX=9940 GN=CS PE=3 SV=1
XP_004006633.1	0.11	20	9	9	Carbamidomethylation	citrate synthase mitochondrial [Ovis aries]
XP_004017458.1	2.63	20	8	8	Carbamidomethylation	aldehyde dehydrogenase mitochondrial [Ovis aries]
XP_027815150.1	2.38	50	8	8	Carbamidomethylation; Deamidation (NQ)	phosphatidylethanolamine-binding protein 1 [Ovis aries]
XP_027817851.1	5.03	77	30	7	Carbamidomethylation; Deamidation (NQ); Oxidation (M); Phosphorylation (STY); Dehydration; Pyro-glu from Q	fructose-bisphosphate aldolase A [Ovis aries]
XP_027815667.1	3.62	44	10	7	Carbamidomethylation; Deamidation (NQ); Oxidation (M); Phosphorylation (STY); Pyro-glu from Q; 2 more	troponin I fast skeletal muscle [Ovis aries]
XP_027824959.1	1.43	30	7	7	Carbamidomethylation; Deamidation (NQ)	voltage-dependent anion-selective channel protein 1 isoform X1 [Ovis aries]
XP_004015238.1	1.05	67	7	7	Carbamidomethylation	cytochrome c oxidase subunit 6B1 [Ovis aries]
XP_027830687.1	0.96	42	135	7	Carbamidomethylation; Deamidation (NQ); Oxidation (M); Phosphorylation (STY); Dehydration; 2 more	myosin-8 [Ovis aries]
XP_027831980.1	0.47	20	7	7	Carbamidomethylation; Deamidation (NQ)	myosin-binding protein H [Ovis aries]
XP_004020879.1	0.23	23	7	7	Carbamidomethylation; Deamidation (NQ); Pyro-glu from Q	cytochrome b-c1 complex subunit 2 mitochondrial [Ovis aries]
XP_027817094.1	0.14	20	7	7	Carbamidomethylation	tripartite motif-containing protein 72 [Ovis aries]
W5Q0R4|W5Q0R4_SHEEP	3.54	37	6	6	Deamidation (NQ); Pyro-glu from Q	Alpha-crystallin B chain OS=Ovis aries OX=9940 GN=CRYAB PE=3 SV=1
W5PF65|W5PF65_SHEEP	3.17	9	6	6	Carbamidomethylation	Transferrin OS=Ovis aries OX=9940 GN=TF PE=3 SV=1
XP_027816111.1	3.17	9	6	6	Carbamidomethylation	serotransferrin [Ovis aries]

XP_027821559.1	3.13	48	6	6	Carbamidomethylation; Deamidation (NQ)	galectin-1 isoform X1 [Ovis aries]
W5PWZ2|W5PWZ2_SHEEP	3.13	60	6	6	Carbamidomethylation; Deamidation (NQ)	Galectin OS=Ovis aries OX=9940 GN=LGALS1 PE=4 SV=1
W5PFT7|W5PFT7_SHEEP	3.01	35	6	6	Carbamidomethylation; Deamidation (NQ)	Fructose-bisphosphatase 2 OS=Ovis aries OX=9940 GN=FBP2 PE=3 SV=1
NP_001119823.1	2.79	71	32	6	Carbamidomethylation; Deamidation (NQ); Oxidation (M); Phosphorylation (STY); Dehydration; 2 more	tropomyosin alpha-1 chain [Ovis aries]
W5Q8N4|W5Q8N4_SHEEP	2.19	51	6	6	Deamidation (NQ)	Myosin light chain 2 OS=Ovis aries OX=9940 GN=MYL2 PE=2 SV=1
W5PP37|W5PP37_SHEEP	1.55	45	6	6	Carbamidomethylation	ATP synthase subunit d mitochondrial OS=Ovis aries OX=9940 GN=ATP5PD PE=3 SV=1
NP_001136363.1	1.55	45	6	6	Carbamidomethylation	ATP synthase subunit d mitochondrial [Ovis aries]
XP_027820491.1	1.54	17	6	6	Deamidation (NQ)	myc box-dependent-interacting protein 1 isoform X10 [Ovis aries]
XP_027820483.1	1.54	15	6	6	Deamidation (NQ)	myc box-dependent-interacting protein 1 isoform X2 [Ovis aries]
XP_027820501.1	1.54	20	6	6	Deamidation (NQ)	myc box-dependent-interacting protein 1 isoform X20 [Ovis aries]
XP_027820492.1	1.54	18	6	6	Deamidation (NQ)	myc box-dependent-interacting protein 1 isoform X11 [Ovis aries]
XP_027820498.1	1.54	19	6	6	Deamidation (NQ)	myc box-dependent-interacting protein 1 isoform X17 [Ovis aries]
XP_027820488.1	1.54	17	6	6	Deamidation (NQ)	myc box-dependent-interacting protein 1 isoform X7 [Ovis aries]
XP_027820484.1	1.54	16	6	6	Deamidation (NQ)	myc box-dependent-interacting protein 1 isoform X3 [Ovis aries]
XP_027820482.1	1.54	15	6	6	Deamidation (NQ)	myc box-dependent-interacting protein 1 isoform X1 [Ovis aries]
XP_027820499.1	1.54	19	6	6	Deamidation (NQ)	myc box-dependent-interacting protein 1 isoform X18 [Ovis aries]
XP_027820489.1	1.54	17	6	6	Deamidation (NQ)	myc box-dependent-interacting protein 1 isoform X8 [Ovis aries]
XP_027820495.1	1.54	18	6	6	Deamidation (NQ)	myc box-dependent-interacting protein 1 isoform X14 [Ovis aries]
XP_027820496.1	1.54	18	6	6	Deamidation (NQ)	myc box-dependent-interacting protein 1 isoform X15 [Ovis aries]
XP_027820490.1	1.54	17	6	6	Deamidation (NQ)	myc box-dependent-interacting protein 1 isoform X9 [Ovis aries]

XP_027820485.1	1.54	16	6	6	Deamidation (NQ)	myc box-dependent-interacting protein 1 isoform X4 [Ovis aries]
XP_027820504.1	1.54	21	6	6	Deamidation (NQ)	myc box-dependent-interacting protein 1 isoform X23 [Ovis aries]
XP_027820497.1	1.54	19	6	6	Deamidation (NQ)	myc box-dependent-interacting protein 1 isoform X16 [Ovis aries]
XP_027820502.1	1.54	20	6	6	Deamidation (NQ)	myc box-dependent-interacting protein 1 isoform X21 [Ovis aries]
XP_027820493.1	1.54	18	6	6	Deamidation (NQ)	myc box-dependent-interacting protein 1 isoform X12 [Ovis aries]
XP_027820487.1	1.54	17	6	6	Deamidation (NQ)	myc box-dependent-interacting protein 1 isoform X6 [Ovis aries]
XP_027820503.1	1.54	21	6	6	Deamidation (NQ)	myc box-dependent-interacting protein 1 isoform X22 [Ovis aries]
XP_027820494.1	1.54	18	6	6	Deamidation (NQ)	myc box-dependent-interacting protein 1 isoform X13 [Ovis aries]
XP_027820500.1	1.54	20	6	6	Deamidation (NQ)	myc box-dependent-interacting protein 1 isoform X19 [Ovis aries]
XP_027820594.1	1.11	28	754	6	Carbamidomethylation; Deamidation (NQ); Oxidation (M); Phosphorylation (STY); Dehydration; 3 more	LOW QUALITY PROTEIN: titin [Ovis aries]
XP_004010842.2	0.93	14	6	6		dihydrolipoyllysine-residue succinyltransferase component of 2-oxoglutarate dehydrogenase complex mitochondrial [Ovis aries]
W5NTS6|W5NTS6_SHEEP	0.93	14	6	6		Dihydrolipoamide S-succinyltransferase OS=Ovis aries OX=9940 GN=DLST PE=4 SV=1
XP_027829100.1	0.9	1	6	6		plectin isoform X2 [Ovis aries]
XP_027829099.1	0.9	1	6	6		plectin isoform X1 [Ovis aries]
XP_027829103.1	0.9	1	6	6		plectin isoform X4 [Ovis aries]
XP_027829102.1	0.9	1	6	6		plectin isoform X3 [Ovis aries]
W5QFQ1|W5QFQ1_SHEEP	0.71	22	6	6	Carbamidomethylation; Deamidation (NQ)	Malate dehydrogenase OS=Ovis aries OX=9940 GN=MDH1 PE=3 SV=1
W5QFQ0|W5QFQ0_SHEEP	0.71	23	6	6	Carbamidomethylation; Deamidation (NQ)	Malate dehydrogenase OS=Ovis aries OX=9940 GN=MDH1 PE=3 SV=1
XP_004005894.2	0.71	23	6	6	Carbamidomethylation; Deamidation (NQ)	malate dehydrogenase cytoplasmic [Ovis aries]

XP_027830504.1	0.66	24	6	6	Deamidation (NQ)	tropomodulin-4 [Ovis aries]
NP_001138655.1	0.64	85	36	6	Carbamidomethylation; Deamidation (NQ); Oxidation (M); Phosphorylation (STY); Dehydration; 3 more	myosin regulatory light chain 2 skeletal muscle isoform [Ovis aries]
XP_004022002.1	4.45	24	5	5	Carbamidomethylation; Deamidation (NQ)	pyruvate dehydrogenase E1 component subunit alpha somatic form mitochondrial [Ovis aries]
W5PIG6|W5PIG6_SHEEP	4.31	40	15	5	Carbamidomethylation; Deamidation (NQ); Oxidation (M); Phosphorylation (STY); Dehydration	Enolase 1 OS=Ovis aries OX=9940 GN=ENO1 PE=3 SV=1
XP_027831475.1	4.31	42	15	5	Carbamidomethylation; Deamidation (NQ); Oxidation (M); Phosphorylation (STY); Dehydration	alpha-enolase isoform X2 [Ovis aries]
XP_004002406.2	3.43	7	5	5		AMP deaminase 1 isoform X1 [Ovis aries]
XP_004002407.1	3.43	7	5	5		AMP deaminase 1 isoform X2 [Ovis aries]
W5QFJ2|W5QFJ2_SHEEP	3.43	7	5	5		AMP deaminase OS=Ovis aries OX=9940 GN=AMPD1 PE=3 SV=1
W5PNI5|W5PNI5_SHEEP	3.28	8	16	5	Carbamidomethylation; Deamidation (NQ); Formylation	Myosin heavy chain 7B OS=Ovis aries OX=9940 GN=MYH7B PE=3 SV=1
sp|P62896|CYC_SHEEP	2.02	50	6	5		Cytochrome c OS=Ovis aries OX=9940 GN=CYCS PE=1 SV=2
XP_011982042.2	1.54	22	5	5	Pyro-glu from Q	peroxiredoxin-1 [Ovis aries]
XP_004002090.1	1.06	66	31	5	Carbamidomethylation; Deamidation (NQ); Oxidation (M); Pyro-glu from Q	phosphoglucomutase-1 isoform X2 [Ovis aries]
XP_004007775.1	1.04	4	5	5		collagen alpha-2(I) chain [Ovis aries]
XP_027812976.1	0.91	54	5	5	Deamidation (NQ)	cytochrome c oxidase subunit 5A mitochondrial [Ovis aries]
W5NXT8|W5NXT8_SHEEP	0.91	68	5	5	Deamidation (NQ)	Cytochrome c oxidase subunit 5A OS=Ovis aries OX=9940 GN=COX5A PE=1 SV=1
XP_004013798.1	0.57	38	5	5	Carbamidomethylation	protein/nucleic acid deglycase DJ-1 [Ovis aries]
sp|P02190|MYG_SHEEP	5.91	62	16	4	Deamidation (NQ); Oxidation (M); Dehydration; Formylation	Myoglobin OS=Ovis aries OX=9940 GN=MB PE=1 SV=2
XP_027818348.1	4.14	18	4	4	Carbamidomethylation	PDZ and LIM domain protein 3 isoform X2 [Ovis aries]

XP_027827422.1	2.47	37	89	4	Carbamidomethylation; Deamidation (NQ); Oxidation (M); Phosphorylation (STY); Dehydration; 2 more	myosin-6 isoform X2 [Ovis aries]
A0A1X9H6E5|A0A1X9H6E5_SHEEP	2.32	21	4	4	Carbamidomethylation	Troponin I type 1 variant X2 OS=Ovis aries OX=9940 GN=TNNI1 PE=2 SV=1
XP_027831854.1	2.32	22	4	4	Carbamidomethylation	troponin I slow skeletal muscle [Ovis aries]
W5PEA4|W5PEA4_SHEEP	1.89	8	4	4	Carbamidomethylation	Succinate--CoA ligase [ADP-forming] subunit beta mitochondrial OS=Ovis aries OX=9940 GN=SUCLA2 PE=3 SV=1
W5PEA2|W5PEA2_SHEEP	1.89	7	4	4	Carbamidomethylation	Succinate--CoA ligase [ADP-forming] subunit beta mitochondrial OS=Ovis aries OX=9940 GN=SUCLA2 PE=3 SV=1
XP_027829360.1	1.89	8	4	4	Carbamidomethylation	succinate--CoA ligase [ADP-forming] subunit beta mitochondrial [Ovis aries]
W5PH95|W5PH95_SHEEP	1.18	21	4	4	Carbamidomethylation; Deamidation (NQ)	Uncharacterized protein OS=Ovis aries OX=9940 PE=4 SV=1
XP_027817699.1	0.98	6	4	4		sarcalumenin isoform X1 [Ovis aries]
W5NYM5|W5NYM5_SHEEP	0.98	6	4	4		Sarcalumenin OS=Ovis aries OX=9940 GN=SRL PE=3 SV=1
XP_004021201.1	0.98	11	4	4		sarcalumenin isoform X2 [Ovis aries]
XP_004007802.1	0.82	46	4	4		cytochrome c oxidase subunit NDUFA4 [Ovis aries]
NP_001091117.1	0.79	86	12	4	Carbamidomethylation; Deamidation (NQ)	hemoglobin subunit beta [Ovis aries]
XP_012038833.2	0.52	18	4	4	Carbamidomethylation; Deamidation (NQ)	cytochrome c1 heme protein mitochondrial [Ovis aries]
NP_001138659.1	0.45	59	4	4	Carbamidomethylation	cytochrome b-c1 complex subunit 6 mitochondrial [Ovis aries]
W5NPN4|W5NPN4_SHEEP	0.26	18	9	4	Carbamidomethylation; Deamidation (NQ)	Heat shock protein family A (Hsp70) member 8 OS=Ovis aries OX=9940 GN=HSPA8 PE=3 SV=1
XP_011951023.2	0.26	18	9	4	Carbamidomethylation; Deamidation (NQ)	heat shock cognate 71 kDa protein [Ovis aries]
NP_001155363.1	0.26	15	4	4	Deamidation (NQ)	fumarate hydratase mitochondrial [Ovis aries]
XP_027833508.1	0.01	20	4	4		cytochrome c oxidase subunit 4 isoform 1 mitochondrial [Ovis aries]

W5PPE8|W5PPE8_SHEEP	0.01	20	4	4		Cytochrome c oxidase subunit 4I1 OS=Ovis aries OX=9940 GN=COX4I1 PE=1 SV=1
W5PAN7|W5PAN7_SHEEP	0	33	6	4	Carbamidomethylation; Deamidation (NQ); Dehydration	Myosin light chain 3 OS=Ovis aries OX=9940 GN=MYL3 PE=4 SV=1
XP_027813530.1	0	33	6	4	Carbamidomethylation; Deamidation (NQ); Dehydration	myosin light chain 3 [Ovis aries]
XP_027836013.1	14.12	9	3	3	Carbamidomethylation; Deamidation (NQ)	succinate dehydrogenase [ubiquinone] flavoprotein subunit mitochondrial [Ovis aries]
W5Q216|W5Q216_SHEEP	14.12	9	3	3	Carbamidomethylation; Deamidation (NQ)	Succinate dehydrogenase [ubiquinone] flavoprotein subunit mitochondrial OS=Ovis aries OX=9940 PE=3 SV=1
XP_011950844.2	4.38	10	3	3	Carbamidomethylation; Deamidation (NQ)	dihydrolipoyllysine-residue acetyltransferase component of pyruvate dehydrogenase complex mitochondrial [Ovis aries]
W5Q2C5|W5Q2C5_SHEEP	4.38	10	3	3	Carbamidomethylation; Deamidation (NQ)	Acetyltransferase component of pyruvate dehydrogenase complex OS=Ovis aries OX=9940 PE=3 SV=1
XP_004008028.1	4.37	3	3	3		2-oxoglutarate dehydrogenase mitochondrial isoform X4 [Ovis aries]
XP_004008027.1	4.37	3	3	3		2-oxoglutarate dehydrogenase mitochondrial isoform X3 [Ovis aries]
XP_012032355.1	4.37	3	3	3		2-oxoglutarate dehydrogenase mitochondrial isoform X2 [Ovis aries]
XP_012032354.1	4.37	3	3	3		2-oxoglutarate dehydrogenase mitochondrial isoform X1 [Ovis aries]
XP_027832746.1	3.91	15	5	3	Deamidation (NQ)	elongation factor 1-alpha 2 [Ovis aries]
W5PN24|W5PN24_SHEEP	3.91	14	5	3	Deamidation (NQ)	Eukaryotic translation elongation factor 1 alpha 2 OS=Ovis aries OX=9940 GN=EEF1A2 PE=3 SV=1
XP_004008889.1	3.68	6	3	3		stress-70 protein mitochondrial [Ovis aries]
XP_027813217.1	3.3	4	3	3		heat shock protein HSP 90-alpha [Ovis aries]
XP_004009741.1	2.15	14	3	3		PDZ and LIM domain protein 5 isoform X7 [Ovis aries]
XP_027826671.1	2.15	7	3	3		PDZ and LIM domain protein 5 isoform X4 [Ovis aries]
XP_004009740.1	2.15	13	3	3		PDZ and LIM domain protein 5 isoform X6 [Ovis aries]

XP_004009744.1	2.15	6	3	3		PDZ and LIM domain protein 5 isoform X3 [Ovis aries]
XP_004009748.1	2.15	6	3	3		PDZ and LIM domain protein 5 isoform X2 [Ovis aries]
XP_004009739.1	2.15	5	3	3		PDZ and LIM domain protein 5 isoform X1 [Ovis aries]
XP_004021390.1	1.87	76	54	3	Carbamidomethylation; Deamidation (NQ); Oxidation (M); Phosphorylation (STY); Dehydration; 3 more	actin alpha skeletal muscle isoform X4 [Ovis aries]
XP_027813246.1	1.61	22	7	3	Carbamidomethylation; Deamidation (NQ); Phosphorylation (STY); Formylation	creatine kinase B-type [Ovis aries]
XP_011959304.1	1.47	99	33	3	Carbamidomethylation; Deamidation (NQ); Oxidation (M); Dehydration; Pyro-glu from Q; 2 more	myosin regulatory light chain 2 skeletal muscle isoform isoform X1 [Ovis aries]
XP_027830685.1	1.02	61	199	3	Carbamidomethylation; Deamidation (NQ); Oxidation (M); Phosphorylation (STY); Dehydration; 3 more	LOW QUALITY PROTEIN: myosin-2 [Ovis aries]
NP_001120750.1	0.92	29	10	3	Deamidation (NQ)	ADP/ATP translocase 1 [Ovis aries]
XP_014958051.2	0.76	9	3	3		uncharacterized protein LOC101108868 [Ovis aries]
XP_027820543.1	0.76	14	3	3		uncharacterized protein LOC114113063 [Ovis aries]
XP_027814900.1	0.76	9	3	3		uncharacterized protein LOC101120961 [Ovis aries]
W5QFN7|W5QFN7_SHEEP	0.76	27	3	3		Histone H4 OS=Ovis aries OX=9940 PE=3 SV=1
W5QFN8|W5QFN8_SHEEP	0.76	27	3	3		Histone H4 OS=Ovis aries OX=9940 PE=3 SV=1
W5QFP4|W5QFP4_SHEEP	0.76	28	3	3		Histone H4 OS=Ovis aries OX=9940 PE=3 SV=1
W5PHH3|W5PHH3_SHEEP	0.76	28	3	3		Histone H4 OS=Ovis aries OX=9940 PE=3 SV=1
W5PHL0|W5PHL0_SHEEP	0.76	28	3	3		Histone H4 OS=Ovis aries OX=9940 PE=3 SV=1
W5QFR6|W5QFR6_SHEEP	0.76	28	3	3		Histone H4 OS=Ovis aries OX=9940 PE=3 SV=1
W5PHM4|W5PHM4_SHEEP	0.76	27	3	3		Histone H4 OS=Ovis aries OX=9940 GN=LOC101123670 PE=3 SV=1
W5QFN3|W5QFN3_SHEEP	0.76	27	3	3		Histone H4 OS=Ovis aries OX=9940 PE=3 SV=1

XP_004008824.1	0.6	38	3	3		ATP synthase subunit delta mitochondrial [Ovis aries]
W5P642|W5P642_SHEEP	0.36	26	3	3		Cytochrome b-c1 complex subunit 7 OS=Ovis aries OX=9940 GN=UQCRB PE=1 SV=1
XP_027829005.1	0.36	25	3	3		cytochrome b-c1 complex subunit 7 isoform X1 [Ovis aries]
XP_004002472.1	0.35	20	3	3		histone H2B type 2-F [Ovis aries]
XP_004019125.1	0.35	20	3	3		histone H2B type 1-M [Ovis aries]
XP_004019076.1	0.35	20	3	3		histone H2B type 1-N [Ovis aries]
XP_027814886.1	0.35	20	3	3		histone H2B type 1-K [Ovis aries]
W5NRX3|W5NRX3_SHEEP	0.35	19	3	3		Histone H2B OS=Ovis aries OX=9940 PE=3 SV=1
W5NS71|W5NS71_SHEEP	0.35	18	3	3		Histone H2B OS=Ovis aries OX=9940 PE=3 SV=1
W5Q9Y2|W5Q9Y2_SHEEP	0.35	18	3	3		Histone H2B OS=Ovis aries OX=9940 PE=3 SV=1
W5NR98|W5NR98_SHEEP	0.35	18	3	3		Histone H2B OS=Ovis aries OX=9940 PE=3 SV=1
W5QAH2|W5QAH2_SHEEP	0.35	18	3	3		Histone H2B OS=Ovis aries OX=9940 GN=LOC101119426 PE=3 SV=1
W5NR06|W5NR06_SHEEP	0.35	18	3	3		Histone H2B OS=Ovis aries OX=9940 PE=3 SV=1
W5QA24|W5QA24_SHEEP	0.35	18	3	3		Histone H2B OS=Ovis aries OX=9940 PE=3 SV=1
W5QAW7|W5QAW7_SHEEP	0.35	18	3	3		Histone H2B OS=Ovis aries OX=9940 PE=3 SV=1
XP_004019124.2	0.35	17	3	3		histone H2B type 1 [Ovis aries]
W5QC76|W5QC76_SHEEP	0.33	2	6	3	Carbamidomethylation	Myosin heavy chain 15 OS=Ovis aries OX=9940 GN=MYH15 PE=3 SV=1
XP_027817063.1	0.29	4	3	3		collagen alpha-2(VI) chain isoform X1 [Ovis aries]
XP_004005716.1	0.22	20	3	3	Carbamidomethylation; Deamidation (NQ)	NADH dehydrogenase [ubiquinone] 1 alpha subcomplex subunit 8 [Ovis aries]

XP_027820863.1	0.15	32	3	3		10 kDa heat shock protein mitochondrial isoform X2 [Ovis aries]
W5Q1T4|W5Q1T4_SHEEP	0.15	32	3	3		Uncharacterized protein OS=Ovis aries OX=9940 PE=3 SV=1
A0A0H3V384|A0A0H3V384_SHEEP	5.64	78	20	2	Carbamidomethylation; Deamidation (NQ); Oxidation (M); Phosphorylation (STY); Dehydration; Formylation	Myosin light chain 1 OS=Ovis aries OX=9940 GN=MYL1 PE=2 SV=1
XP_004007919.2	5.04	13	2	2		F-actin-capping protein subunit alpha-2 [Ovis aries]
W5PCG7|W5PCG7_SHEEP	4.19	14	2	2		Uncharacterized protein OS=Ovis aries OX=9940 GN=MYOZ3 PE=4 SV=1
XP_027823071.1	3.03	1	2	2		LOW QUALITY PROTEIN: collagen alpha-3(VI) chain [Ovis aries]
W5QCP9|W5QCP9_SHEEP	3.03	1	2	2		Collagen type VI alpha 3 chain OS=Ovis aries OX=9940 GN=COL6A3 PE=4 SV=1
XP_027814043.1	2.9	8	2	2	Carbamidomethylation; Deamidation (NQ)	pyruvate dehydrogenase E1 component subunit beta mitochondrial [Ovis aries]
W5PRM8|W5PRM8_SHEEP	2.9	8	2	2	Carbamidomethylation; Deamidation (NQ)	Pyruvate dehydrogenase E1 component subunit beta OS=Ovis aries OX=9940 PE=4 SV=1
W5PTW1|W5PTW1_SHEEP	2.51	12	2	2	Carbamidomethylation	Uncharacterized protein OS=Ovis aries OX=9940 PE=3 SV=1
XP_027815272.1	2.51	12	2	2	Carbamidomethylation	glutathione S-transferase P [Ovis aries]
W5NQP9|W5NQP9_SHEEP	2.38	15	6	2	Carbamidomethylation; Deamidation (NQ)	Fructose-bisphosphate aldolase OS=Ovis aries OX=9940 GN=ALDOC PE=3 SV=1
XP_014954022.2	2.38	19	6	2	Carbamidomethylation; Deamidation (NQ)	fructose-bisphosphate aldolase C [Ovis aries]
XP_011954300.1	2.16	11	7	2	Carbamidomethylation; Deamidation (NQ); Oxidation (M); Phosphorylation (STY); Formylation	creatine kinase U-type mitochondrial [Ovis aries]
XP_027829423.1	1.93	12	2	2	Deamidation (NQ)	ubiquitin-40S ribosomal protein S27a [Ovis aries]
sp|P0C276|RL40_SHEEP	1.93	20	2	2	Deamidation (NQ)	Ubiquitin-60S ribosomal protein L40 OS=Ovis aries OX=9940 GN=UBA52 PE=2 SV=2
W5Q9W5|W5Q9W5_SHEEP	1.93	5	2	2	Deamidation (NQ)	Ubiquitin C OS=Ovis aries OX=9940 GN=UBC PE=4 SV=1
XP_027812205.1	1.93	3	2	2	Deamidation (NQ)	LOW QUALITY PROTEIN: polyubiquitin-C [Ovis aries]

XP_004005993.3	1.93	16	2	2	Deamidation (NQ)	ubiquitin-40S ribosomal protein S27a [Ovis aries]
sp|P0CG55|UBB_SHEEP	1.93	8	2	2	Deamidation (NQ)	Polyubiquitin-B OS=Ovis aries OX=9940 GN=UBB PE=2 SV=1
sp|P09670|SODC_SHEEP	1.64	24	2	2	Deamidation (NQ)	Superoxide dismutase [Cu-Zn] OS=Ovis aries OX=9940 GN=SOD1 PE=1 SV=2
NP_001138657.1	1.64	24	2	2	Deamidation (NQ)	superoxide dismutase [Cu-Zn] [Ovis aries]
W5QBF5|W5QBF5_SHEEP	1.56	10	2	2		NADH dehydrogenase [ubiquinone] 1 alpha subcomplex subunit 10 mitochondrial OS=Ovis aries OX=9940 GN=NDUFA10 PE=1 SV=1
XP_004001808.2	1.56	10	2	2		NADH dehydrogenase [ubiquinone] 1 alpha subcomplex subunit 10 mitochondrial isoform X1 [Ovis aries]
XP_027822837.1	1.56	11	2	2		NADH dehydrogenase [ubiquinone] 1 alpha subcomplex subunit 10 mitochondrial isoform X2 [Ovis aries]
W5PEL7|W5PEL7_SHEEP	1.4	1	2	2		Heparan sulfate proteoglycan 2 OS=Ovis aries OX=9940 GN=HSPG2 PE=4 SV=1
XP_027821348.1	1.4	1	2	2		LOW QUALITY PROTEIN: basement membrane-specific heparan sulfate proteoglycan core protein [Ovis aries]
XP_014950281.1	1.19	17	3	2	Carbamidomethylation	myosin light chain 6B [Ovis aries]
XP_027812629.1	1.06	35	5	2	Carbamidomethylation; Deamidation (NQ)	immunoglobulin lambda-1 light chain-like [Ovis aries]
XP_027835867.1	0.98	31	17	2	Carbamidomethylation; Deamidation (NQ); Oxidation (M); Phosphorylation (STY); Dehydration; 2 more	beta-actin-like protein 2 isoform X2 [Ovis aries]
XP_012033947.2	0.96	5	2	2	Carbamidomethylation	myotilin isoform X2 [Ovis aries]
W5Q1Q5|W5Q1Q5_SHEEP	0.96	5	2	2	Carbamidomethylation	Myotilin OS=Ovis aries OX=9940 GN=MYOT PE=4 SV=1
XP_004008869.2	0.96	5	2	2	Carbamidomethylation	myotilin isoform X1 [Ovis aries]
XP_027834603.1	0.91	14	3	2		troponin T slow skeletal muscle isoform X5 [Ovis aries]
XP_014955600.2	0.91	14	3	2		troponin T slow skeletal muscle isoform X4 [Ovis aries]
XP_011950163.2	0.91	14	3	2		troponin T slow skeletal muscle isoform X3 [Ovis aries]

XP_027834602.1	0.91	14	3	2		troponin T slow skeletal muscle isoform X2 [Ovis aries]
W5NUR7|W5NUR7_SHEEP	0.91	13	3	2		Troponin T1 slow skeletal type OS=Ovis aries OX=9940 GN=TNNT1 PE=4 SV=1
XP_027834601.1	0.91	13	3	2		troponin T slow skeletal muscle isoform X1 [Ovis aries]
A0A1X9H6F1|A0A1X9H6F1_SHEEP	0.91	13	3	2		Troponin T type 1 variant X1 OS=Ovis aries OX=9940 GN=TNNT1 PE=2 SV=1
XP_004014229.1	0.79	8	2	2		ATP synthase subunit gamma mitochondrial isoform X1 [Ovis aries]
W5P340|W5P340_SHEEP	0.74	13	2	2		Superoxide dismutase OS=Ovis aries OX=9940 PE=3 SV=1
NP_001267632.1	0.74	13	2	2		superoxide dismutase [Mn] mitochondrial [Ovis aries]
W5PUX0|W5PUX0_SHEEP	0.71	8	2	2	Deamidation (NQ)	NADH dehydrogenase [ubiquinone] flavoprotein 1 mitochondrial OS=Ovis aries OX=9940 GN=NDUFV1 PE=1 SV=1
XP_027815074.1	0.71	8	2	2	Deamidation (NQ)	NADH dehydrogenase [ubiquinone] flavoprotein 1 mitochondrial [Ovis aries]
XP_027816001.1	0.66	23	2	2		cytochrome c oxidase subunit 5B mitochondrial [Ovis aries]
W5PSQ7|W5PSQ7_SHEEP	0.52	30	5	2	Carbamidomethylation; Deamidation (NQ)	Uncharacterized protein OS=Ovis aries OX=9940 PE=4 SV=1
XP_004021550.1	0.37	7	2	2		voltage-dependent anion-selective channel protein 2 [Ovis aries]
W5QGQ8|W5QGQ8_SHEEP	0.35	7	2	2	Carbamidomethylation	Succinate--CoA ligase [ADP/GDP-forming] subunit alpha mitochondrial OS=Ovis aries OX=9940 GN=SUCLG1 PE=3 SV=1
XP_004007304.4	0.35	8	2	2	Carbamidomethylation	succinate--CoA ligase [ADP/GDP-forming] subunit alpha mitochondrial [Ovis aries]
XP_014957448.2	0.31	5	2	2		isocitrate dehydrogenase [NAD] subunit alpha mitochondrial [Ovis aries]
W5NR14|W5NR14_SHEEP	0.31	5	2	2		Isocitrate dehydrogenase [NAD] subunit mitochondrial OS=Ovis aries OX=9940 GN=IDH3A PE=3 SV=1
XP_004014945.1	0.25	8	2	2		cytochrome b-c1 complex subunit Rieske mitochondrial [Ovis aries]
XP_004013127.3	0.24	47	33	2	Carbamidomethylation; Deamidation (NQ); Oxidation (M); Phosphorylation (STY); Dehydration; 2 more	actin cytoplasmic 2 [Ovis aries]

sp|P60713|ACTB_SHEEP	0.24	47	33	2	Carbamidomethylation; Deamidation (NQ); Oxidation (M); Phosphorylation (STY); Dehydration; 2 more	Actin cytoplasmic 1 OS=Ovis aries OX=9940 GN=ACTB PE=2 SV=1
XP_027834968.1	0.23	7	2	2		acetyl-CoA acetyltransferase mitochondrial [Ovis aries]
W5PN85|W5PN85_SHEEP	0.23	7	2	2		Acetyl-CoA acetyltransferase 1 OS=Ovis aries OX=9940 GN=ACAT1 PE=3 SV=1
XP_027830506.1	0.21	5	7	2		collagen alpha-1(I) chain isoform X1 [Ovis aries]
W5P481|W5P481_SHEEP	0.21	5	7	2		Collagen type I alpha 1 chain OS=Ovis aries OX=9940 GN=COL1A1 PE=4 SV=1
XP_004012685.1	0.18	4	2	2	Deamidation (NQ)	very long-chain specific acyl-CoA dehydrogenase mitochondrial [Ovis aries]
XP_004007902.1	0.16	5	2	2		dihydrolipoyl dehydrogenase mitochondrial [Ovis aries]
sp|P00922|CAH2_SHEEP	0.13	7	2	2		Carbonic anhydrase 2 OS=Ovis aries OX=9940 GN=CA2 PE=1 SV=2
XP_027829052.1	0.13	7	2	2		carbonic anhydrase 2 [Ovis aries]
W5PTU7|W5PTU7_SHEEP	0.13	6	2	2		Carbonic anhydrase 2 OS=Ovis aries OX=9940 GN=CA2 PE=3 SV=1
W5PXG3|W5PXG3_SHEEP	0.11	20	2	2		COX6C domain-containing protein OS=Ovis aries OX=9940 PE=1 SV=1
W5Q754|W5Q754_SHEEP	0.09	29	750	2	Carbamidomethylation; Deamidation (NQ); Oxidation (M); Phosphorylation (STY); Dehydration; 3 more	Titin OS=Ovis aries OX=9940 GN=TTN PE=4 SV=1
W5PTA0|W5PTA0_SHEEP	0.07	12	2	2		Uncharacterized protein OS=Ovis aries OX=9940 GN=ATP5PO PE=3 SV=1
W5PT98|W5PT98_SHEEP	0.07	11	2	2		Uncharacterized protein OS=Ovis aries OX=9940 GN=ATP5PO PE=3 SV=1
XP_027815230.1	0.06	34	14	2	Deamidation (NQ); Oxidation (M); Pyro-glu from Q	troponin T fast skeletal muscle isoform X25 [Ovis aries]
XP_027815256.1	0.06	43	14	2	Deamidation (NQ); Oxidation (M); Pyro-glu from Q	troponin T fast skeletal muscle isoform X50 [Ovis aries]
XP_027815254.1	0.06	43	14	2	Deamidation (NQ); Oxidation (M); Pyro-glu from Q	troponin T fast skeletal muscle isoform X48 [Ovis aries]
XP_027815252.1	0.06	43	14	2	Deamidation (NQ); Oxidation (M); Pyro-glu from Q	troponin T fast skeletal muscle isoform X46 [Ovis aries]
XP_027815248.1	0.06	42	14	2	Deamidation (NQ); Oxidation (M); Pyro-glu from Q	troponin T fast skeletal muscle isoform X43 [Ovis aries]
XP_027815247.1	0.06	41	14	2	Deamidation (NQ); Oxidation (M); Pyro-glu from Q	troponin T fast skeletal muscle isoform X42 [Ovis aries]

XP_027815245.1	0.06	41	14	2	Deamidation (NQ); Oxidation (M); Pyro-glu from Q	troponin T fast skeletal muscle isoform X40 [Ovis aries]
XP_027815241.1	0.06	40	14	2	Deamidation (NQ); Oxidation (M); Pyro-glu from Q	troponin T fast skeletal muscle isoform X36 [Ovis aries]
XP_027815240.1	0.06	40	14	2	Deamidation (NQ); Oxidation (M); Pyro-glu from Q	troponin T fast skeletal muscle isoform X35 [Ovis aries]
XP_027815233.1	0.06	38	14	2	Deamidation (NQ); Oxidation (M); Pyro-glu from Q	troponin T fast skeletal muscle isoform X28 [Ovis aries]
XP_027815232.1	0.06	37	14	2	Deamidation (NQ); Oxidation (M); Pyro-glu from Q	troponin T fast skeletal muscle isoform X27 [Ovis aries]
XP_027815250.1	0.06	36	14	2	Deamidation (NQ); Oxidation (M); Pyro-glu from Q	troponin T fast skeletal muscle isoform X45 [Ovis aries]
XP_027815243.1	0.06	34	14	2	Deamidation (NQ); Oxidation (M); Pyro-glu from Q	troponin T fast skeletal muscle isoform X38 [Ovis aries]
XP_027815239.1	0.06	34	14	2	Deamidation (NQ); Oxidation (M); Pyro-glu from Q	troponin T fast skeletal muscle isoform X34 [Ovis aries]
XP_027815237.1	0.06	34	14	2	Deamidation (NQ); Oxidation (M); Pyro-glu from Q	troponin T fast skeletal muscle isoform X32 [Ovis aries]
XP_027815235.1	0.06	33	14	2	Deamidation (NQ); Oxidation (M); Pyro-glu from Q	troponin T fast skeletal muscle isoform X30 [Ovis aries]

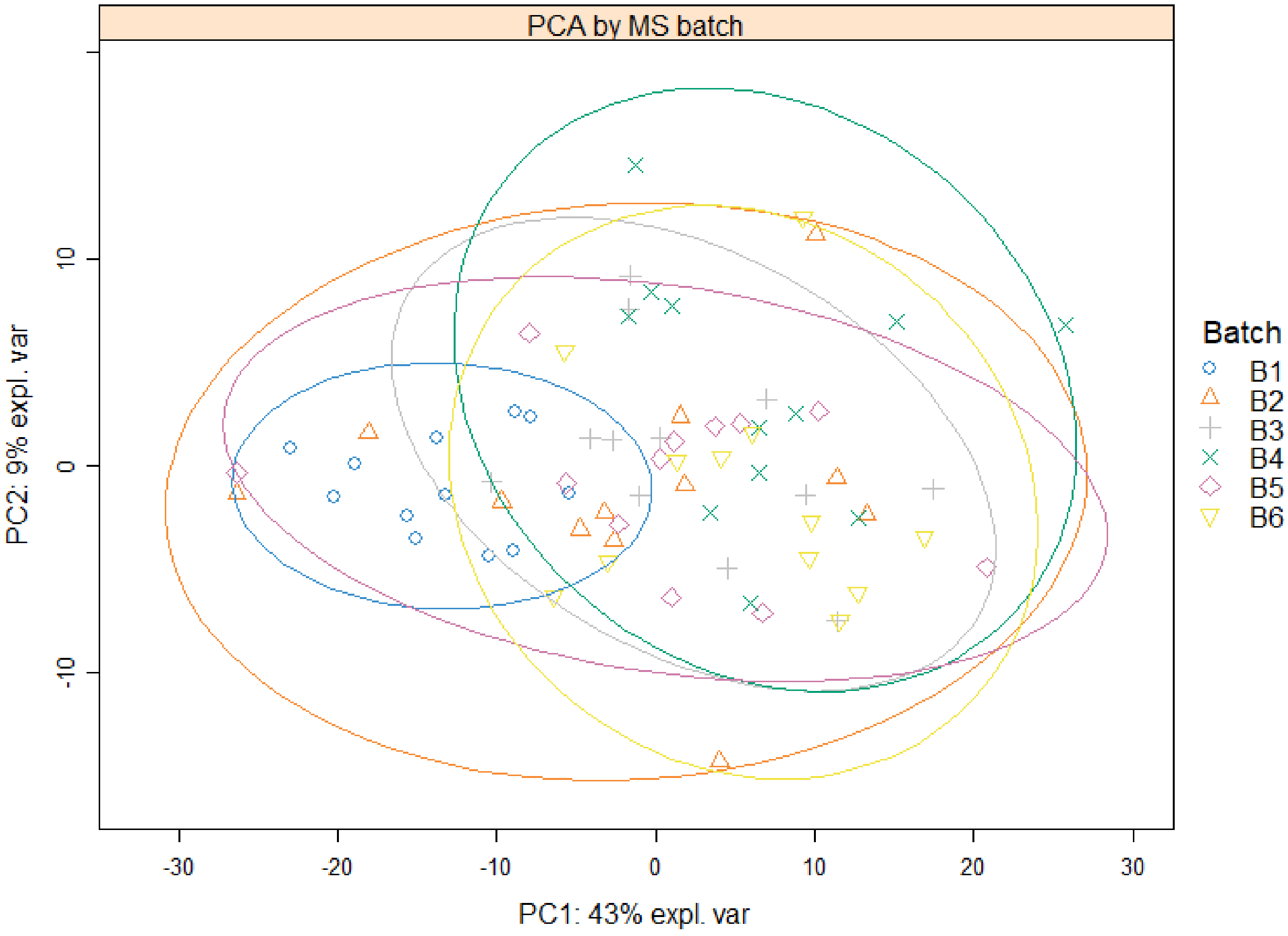

Figure S1. Principal component analysis for each of the six batches of samples from the LC-MSMS analysis. Samples from B1 were identified to have a significant batch effect ($p<0.05$) along the first, second, and third components after performing an analysis of variance on these components.

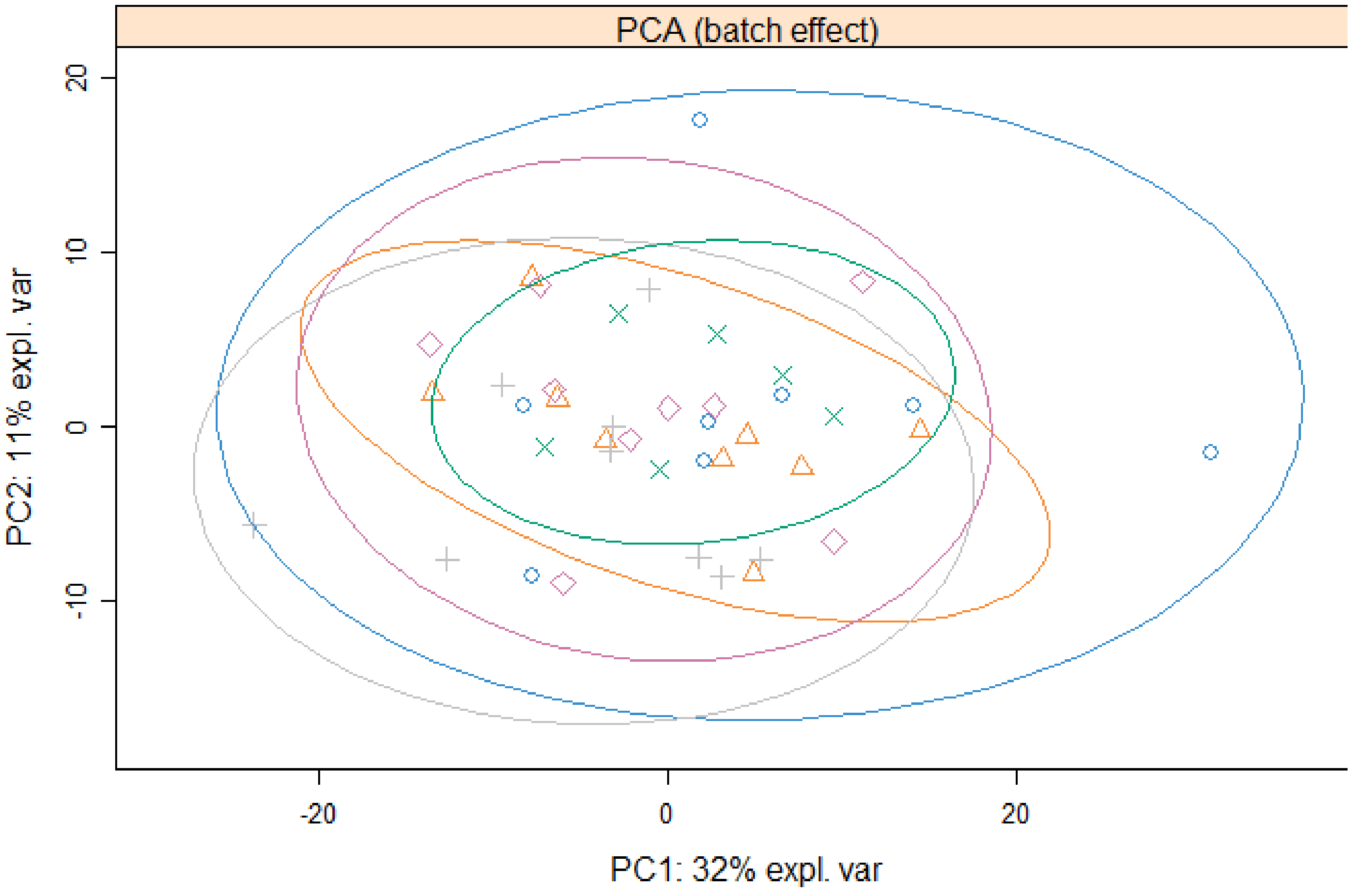

Figure S2. Principal component analysis for the five batches (B2, B3, B4, B5, and B6) of samples from the LC-MSMS analysis after removal of samples from the first batch (B1).
